# Subtypes of nurses’ mental workload and interaction patterns with fatigue and work engagement during coronavirus disease 2019 (COVID-19) outbreak: A latent class analysis

**DOI:** 10.1186/s12912-021-00726-9

**Published:** 2021-10-22

**Authors:** Jing Wu, Husheng Li, Zhaohui Geng, Yanmei Wang, Xian Wang, Jie Zhang

**Affiliations:** 1 School of Nursing, Shanghai University of Traditional Chinese Medicine, 1200 Cai Lun Road, Shanghai, 201203 China; 2 Department of Nursing, Shanghai Municipal Hospital of Traditional Chinese Medicine Affiliated to Shanghai University of TCM, 274 Middle Zhi Jiang Road, Shanghai, 200071 China

**Keywords:** Nurses, Mental workload, Latent class analysis, Fatigue, Work engagement, COVID-19

## Abstract

**Background:**

Nurses play critical roles when providing health care in high-risk situations, such as during the COVID-19 outbreak. However, no previous study had systematically assessed nurses’ mental workloads and its interaction patterns with fatigue, work engagement and COVID-19 exposure risk.

**Methods:**

A cross-sectional study was conducted via online questionnaire. The NASA Task Load Index, Fatigue Scale-14, and Utrecht Work Engagement Scale were used to assess nurses’ mental workload, fatigue and work engagement, respectively. A total of 1337 valid questionnaires were received and analyzed. Nurses were categorized into different subgroups of mental workload via latent class analysis (LCA). Cross-sectional comparisons, analysis of covariance (ANCOVA), and multivariate (or logistic) regression were subsequently performed to examine how demographic variables, fatigue and work engagement differ among nurses belonging to different subgroups.

**Results:**

Three latent classes were identified based on the responses to mental workload assessment: Class 1 – low workload perception & high self-evaluation group (*n* = 41, 3.1%); Class 2 – medium workload perception & medium self-evaluation group (*n* = 455, 34.0%); and Class 3 – high workload perception & low self-evaluation group (*n* = 841, `62.9%). Nurses belonging into class 3 were most likely to be older and have longer professional years, and displayed higher scores of fatigue and work engagement compared with the other latent classes (*p* < 0.05). Multivariate analysis showed that high cognitive workload increased subjective fatigue, and mental workload may be positively associated with work engagement. Group comparison results indicated that COVID-19 exposure contributed to significantly higher mental workload levels.

**Conclusions:**

The complex scenario for the care of patients with infectious diseases, especially during an epidemic, raises the need for improved consideration of nurses’ perceived workload, as well as their physical fatigue, work engagement and personal safety when working in public health emergencies.

## Background

The coronavirus disease 2019 (COVID-19) outbreak caused by severe acute respiratory syndrome coronavirus 2 (SARS-CoV-2) has threatened to overwhelm healthcare systems worldwide [1]. In mainland China, nurses are at the forefront of providing critical healthcare for COVID-19 patients and have been considered the major workforce in pandemic control [2]. Nurses have been inevitably faced with increased psychological distress and physical exhaustion during this time, which may have adverse effects on not only their mental health, but also their level of work engagement and COVID-19 exposure risk. Although the relationship between physical workload and its direct neuropsychiatric consequences has been well established, few studies have focused on examining the combination of these factors, in terms of mental workload in healthcare workers.

Mental workload refers to the amount of mental effort required in relation to a worker’s ability in completing a task, which may be influenced by numerous factors such as task demand, external support, worker’s cognition, and past experience [3, 4]. Mental workload involves subjective processes and can affect physical skills causing fatigue and functional errors, then leading to change behavior and job performance [4]. High mental workload in workers may result in a decreased capacity for attention, and an increased risk of delayed responses, ultimately contributing to workplace errors [5]. Therefore, it is necessary to pay more attention to the medical personnel’s mental workload. Mental workload level is measured by the National Aeronautics and Space Administration Task Load Index (NASA-TLX) and contains a total of six dimensions: mental, physical, and temporal task demands, as well as effort, frustration, and perceived performance [6]. Due to its multidimensional characteristics, measurement of mental workload may provide a more comprehensive understanding of nurses’ work status and performance during the COVID-19 outbreak.

The job demands-resources (JD-R) model proposes that working conditions can be categorized into job demands and job resources, that are differentially related to specific outcomes [7]. Job demands such as high workload results in stress responses, that are posited to be more strongly related to fatigue, whereas resources are related to work engagement [8]. A study among Italian researchers showed that mental workload only indirectly leads to fatigue through stress and emotional dissonance, but rather was highly predictive of the level of work engagement [9]. The relationship between mental workload and work engagement among front-line nurses in Wuhan was recently documented [10], which showed that work engagement was negatively associated with frustration, but positively associated with mental demand and perceived performance. In general, studies investigating workers’ mental workload usually analyzed only a single outcome, thereby limiting the understanding of potential links between mental workload, fatigue and work engagement. Recent evidence from ergonomics research suggests that analysis of mental workload using the NASA-TLX needs to take into account each of its subtypes rather than a global score.

In actuality, characterization of mental workload subtypes and its related factors are rather complicated. Other than fatigue and work engagement, the demographic data may also have potential correlations with mental workload. In the current study, we assessed the exposure risk of COVID-19 as one of the covariables, and hypothesized that the mental workload level and its subtypes may differ according to the risk exposure level of COVID-19 in healthcare workers. Current studies tend to rely on variable-centered methods, which can obscure individual variations in the six dimensions of NASA-TLX, and therefore a “person-centered” approach may be more effective [11, 12]. One of the most popular and useful methods involves latent class analysis (LCA), which can identify subtypes of participants who share similar patterns based on the variables of interest [13].

There are few previous studies to explore whether there exist different mental workload subgroups in nursing, especially during COVID-19 outbreak. The main study objective is to investigate the level of nurses’ mental workload during the COVID-19 outbreak, clarify the subgroups of nurses’ mental workload, and examine how demographic variables, fatigue and work engagement differ among nurses belonging to different subgroups. We hypothesized that nurses can be separated into different subgroups based on the assessment of NASA-TLX, and that key factors including demographic characteristics, fatigue, and work engagement differed across different subgroups. Specifically, we examined the risk of COVID-19 exposure in order to determine the discrepancy in self-perception and perceived workload during this global health crisis. This study is the first of its type that has been conducted in China, and provides an important reference point for comparison with future international studies.

## Methods

### Design

We employed a cross-sectional design using data from an online questionnaire survey which specially designed for this study and aimed for a convenience sample of participants.

### Data sources and sampling

Full-time nurses who worked during the COVID-19 outbreak were invited to complete an anonymous and self-rated questionnaire in March 2020. Nursing students and long-term off-duty nurses were excluded, as they were not at work during the outbreak and cannot accurately reflect the mental workload faced.

All study participants were recruited from 6 Grade-A tertiary hospitals in Shanghai. Respondents included those who have been working in intensive care unit, fever clinic, emergency department, outpatient department, general wards or nursing departments, as well as those who were sent to Wuhan by the hospital for assistance during the outbreak.

Internet questionnaires were distributed to 1551 nurses via Wenjuanxing (*https://www.wjx.cn*), an online survey platform in China, in order to facilitate the study across different hospitals and regions and avoid unnecessary face-to-face contact during the outbreak. Researchers who took part in the data collection process directly or indirectly had no conflicting interests so as to maximize voluntary participation and protect confidentiality. Questionnaires with more than 10% of items left unanswered or containing logical errors were excluded from the study. Finally, a total of 1337 questionnaires were available for further analysis, 214 questionnaires were excluded from the study, the response rate was 86.2%.

### Measures

In order to analyze the mental workload of nurses by socio-demographic characteristics, the baseline characteristics were recorded including sex, age, marital status, parental status, educational level, professional title, and years of nursing experience. Furthermore, COVID-19 work-related information were recorded including degree of exposure to the virus, actual working hours per day, work experience in public health emergencies, and willingness to care for or assist patients during the COVID-19 outbreak. The degree of exposure to the virus is divided into three levels according to the risk of frontline nurses contacting people infected with SARS-CoV-2: nurses who involved in the care of confirmed patients, going to Wuhan for support and working in fever clinics are high exposure levels; nurses in general outpatient and emergency departments or who have a lot of opportunities to contact the crowd are classified as medium exposure; nurses who have contact with limited personnel in general wards, equipment sterilization rooms and management positions etc. are considered to be low exposure risk levels.

We assessed the participants’ mental workload using the NASA Task Load Index (NASA-TLX) [14, 15]. The current version is made up of six items and reflects the mental workload based on two underlying dimensions: workload perception and self-evaluation. Response items including mental, physical and temporal task demands, effort, and frustration, were marked on a scale ranging from 0 (very low) to 20 (very high), as well as performance, which ranged from 0 (perfect) to 20 (failure). By taking a non-weighted (raw) summed numeric score of the items, the overall mental workload was calculated from a minimum of 0, up to a maximum of 120. Higher scores indicated nurses who faced heavier mental workloads during the COVID-19 outbreak. Liang [16] translated the Chinese version of NASA-TLX and demonstrated the scale’s validity and reliability in Chinese nurses, with a Cronbach’s alpha score of 0.782.

The Fatigue Scale-14 (FS-14) [17] was designed to reflect the recent physical fatigue symptoms and severity of participants. The dichotomized survey is comprised of 14 items ranging from measurements of physical fatigue (eight items) and mental distress (six items). Higher scores for a given scale indicated participants who had stronger fatigue. Xu et al. [18] translated the Chinese version of FS-14 and demonstrated the scale’s validity and reliability in Chinese nurses, with a Cronbach’s alpha score of 0.77.

We assessed the participants’ work state via the Utrecht Work Engagement Scale (UWES) [19], which is a self-reporting inventory composed of 17 items based on three components: vigor (six items), dedication (five items), and absorption (six items). Each item was measured on a seven-point Likert scale ranging from 0 (never) to 6 (always). Higher scores indicated nurses who had greater work engagement. Zhang and Gan [20] translated the Chinese version of UWES and demonstrated the scale’s validity and reliability in a Chinese population, with a Cronbach’s alpha score of 0.90.

### Statistical analyses

We performed LCA using Mplus version 7.2, where we explored homogenous subgroups in a heterogeneous group and then observed categorical variables in each subgroup [21]. To determine the most appropriate latent class model, the combination of model fitting indexes was considered, including Akaike information criterion (AIC), Bayesian information criterion (BIC), sample-size adjustment BIC (ABIC), the Lo–Mendell–Rubin likelihood ratio test (LMR-LRT), and entropy, along with the research interpretability. The best-fit model selected was based on the following criteria [22]: (1) LMR-LRT ≤ 0.05; (2) higher entropy value, and lower information criterion score; (3) interpretability.

After examining the best-fit model of latent classes based on nurses’ conditional response of the NASA-TLX, the data was further adjusted by demographic characteristics, compared the score of work engagement and fatigue through covariance analysis (ANCOVA) and multivariate (or logistic) regression across the subgroups. Simultaneously, we used a multiple correspondence analysis (MCA) model to explore the potential relationship between latent classes and various influence factors based on a visualized factor map. Statistical significance of all tests was set as two-sided *P* value < 0.05. All analyses of data were conducted using SPSS statistical version 26.0.

## Results

### Latent classes of nurses’ mental workload response

In order to classify and identify the optimal model, we extracted and compared the model solutions from the two-class to six-class models. The model diagnostics for the overall samples were summarized in Table 1. The five-class and six-class models of LMR-LRT were not significant, and thus neither was considered. Whereas the two-class model had the highest information criterion score and the four-class model had the lowest entropy value, both models had shortcomings. We thus identified that the three-class model for nurses’ mental workload conditional response provided the best overall model fit, including a statistically significant LMR-LRT *p*-value, the best entropy, and relatively lower information criterion scores.

**Table 1:** Latent class model fit index

Model	AIC	BIC	ABIC	LMR-LRT	ALMR-LRT	BLRT	Entropy
2-class LCA	44,084.315	44,183.081	44,122.726	0.0000	0.0000	0.0000	0.839
**3-class LCA**	**43,617.022**	**43,752.175**	**43,669.584**	**0.0412**	**0.0434**	**0.0000**	**0.873**
4-class LCA	43,308.326	43,479.866	43,375.040	0.0060	0.0065	0.0000	0.808
5-class LCA	43,175.542	43,383.470	43,256.407	0.1582	0.1632	0.0000	0.829
6-class LCA	43,029.236	43,273.551	43,124.252	0.4491	0.4562	0.0000	0.840

*AIC* Akaike information criterion, *BIC* Bayesian information criterion, *ABIC* sample-size-adjusted Bayesian information criterion, *LMR-LRT* the Lo–Mendell–Rubin likelihood ratio test, *ALMR-LRT* Lo-Mendell-Rubin adjusted likelihood ratio test, *BLRT* bootstrapped likelihood ratio test

**Fig. 1:**
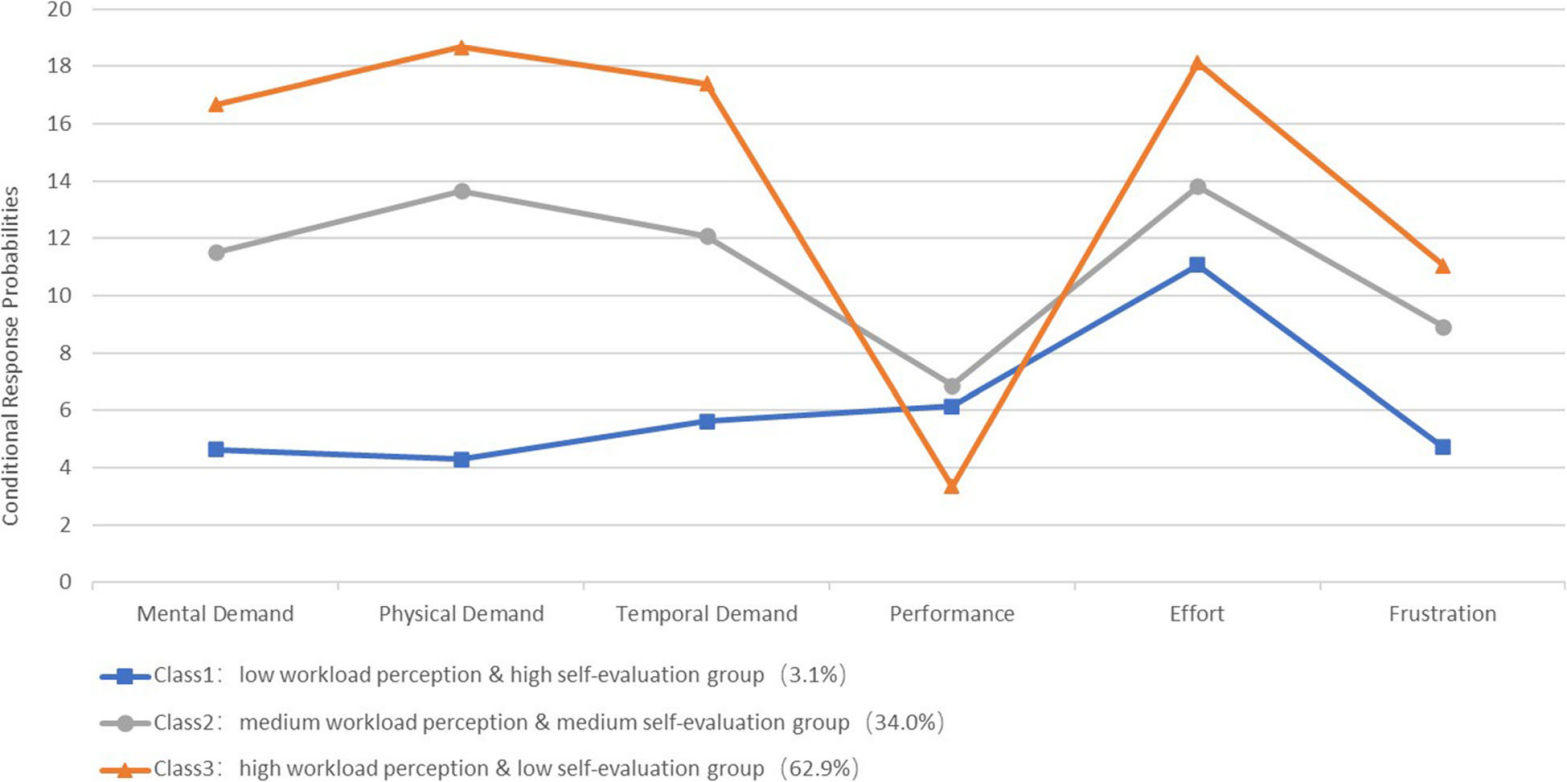
Three subtypes of nurses’ mental workload based on the LCA results

The profiles of the three latent classes based on the LCA results were listed in Table 2, while a line diagram representation was shown in Fig. 1. The x-axis represented six items of NASA-TLX and the y-axis showed the probability of conditional response. Higher levels of mental demand, physical demand, temporal demand, and effort indicated higher perceptive workload, while higher scores of performance and frustration indicated lower self-evaluation. Nurses in class 1 had the lowest workload perception and relatively high self-evaluation, with a total of 3.1% (*n* = 41) of all participants belonging to this group. We therefore classified nurses belonging to class 1 as the “low workload perception & high self-evaluation group”. Nurses in class 2 tended to have average scores of workload perception and self-evaluation, and had no anomalies in each of their mental workload scores. We therefore categorized nurses belonging to class 2 as the “medium workload perception & medium self-evaluation group”, with a total of 34.0% (*n* = 455) of all participants belonging to this group. Nurses in class 3 displayed the highest workload perception and relatively low self-evaluation. Thus, nurses belonging to class 3 were classified as “high workload perception & low self-evaluation group”, which comprised a total of 62.9% (*n* = 841) of all participants.

**Table 2:** The profiles of the classification

Model/Model Mean	Mental Demand	Physical Demand	Temporal Demand	Performance	Effort	Frustration
Class 1 (*n* = 41, 3.1%)	4.622	4.283	5.625	6.148	11.083	4.708
Class 2 (*n* = 455, 34.0%)	11.515	13.659	12.091	6.874	13.810	8.922
Class 3 (*n* = 841, 62.9%)	16.680	18.675	17.407	3.346	18.125	11.057

Class 1: low workload perception & high self-evaluation group; Class 2: medium workload perception & medium self-evaluation group; Class 3: high workload perception & low self-evaluation group

### Differences in the latent classes by characteristics

The statistical description for distributions of each demographic group within latent classes were listed in Table 3. A total of 1337 subjects were enrolled in the study, including 1278 females (95.59%) and 59 males. There were few differences based on sex. Univariate analysis results among the groups showed that participants belonging to class 1 were mostly aged< 28 (56.10%) and have nursing profession< 5 year (46.34%). Class 2 and 3 both had a high proportion of bachelor’s degree or above (57.36 and 63.85%, respectively), with class 2 being younger overall compared to class 3. Participants belonging to class 3 were most aged 28 ~ 38 (41.14%), married (71.22%), have children (63.26%), have nursing profession> 10 years (47.21%), and have high COVID-19 exposure (10.07%, *p* < 0.05 for all).

**Table 3:** Demographic characteristics of mental workload subgroups

	Overall *n* = 1337	Class 1 *n* = 41	Class 2 *n* = 455	Class 3 *n* = 841	*p*
Sex (Female)	1278 (95.59)	40 (97.56)	428 (94.07)	810 (96.31)	0.140
Age (Yrs.)					< 0.001**
< 28	496 (37.10)	23 (56.10)	196 (43.08)	277 (32.94)	
28–38	523 (39.12)	9 (21.95)	168 (36.92)	346 (41.14)	
> 38	318 (23.78)	9 (21.95)	91 (20.00)	218 (25.92)	
Education					0.011*
bachelor’s degree	817 (61.11)	19 (46.34)	261 (57.36)	537 (63.85)	
Non-bachelor’s degree	520 (38.89)	22 (53.66)	194 (42.64)	304 (36.15)	
Nursing profession year					< 0.001**
< 5	419 (31.14)	19 (46.34)	169 (37.14)	231 (27.47)	
5–10	355 (26.55)	8 (19.51)	134 (29.45)	213 (25.33)	
> 10	563 (42.11)	14 (34.15)	152 (33.41)	397 (47.21)	
Marital status (Married)	899 (67.24)	22 (53.66)	278 (61.10)	599 (71.22)	< 0.001**
Child of all ages (Yes)	784 (58.64)	19 (46.34)	233 (51.21)	532 (63.26)	< 0.001**
Degree of Exposure to COVID-19					
Low COV Exp	820 (61.33)	28 (68.29)	276 (60.66)	516 (61.36)	0.016*
Med COV Exp	389 (29.09)	9 (21.95)	145 (31.87)	241 (27.94)	
High COV Exp	128 (9.57)	4 (9.76)	34 (7.47)	90 (10.07)	

Class 1: low workload perception & high self-evaluation group; Class 2: medium workload perception & medium self-evaluation group; Class 3: high workload perception & low self-evaluation group

*Low COV Exp* Low COVID-19 exposure, *Med COV Exp* Medium COVID-19 exposure, *High COV Exp* High COVID-19 exposure

*p < 0.05; **p < 0.01

The mean scores of FS-14 among subjects were 6.94 ± 2.43, and UWES were 57.75 ± 11.47, as shown in Table 4. There were significant differences among the three interaction classes, including the scores of FS-14, UWES, and their dimension scores (*p* < 0.05 for all), except for the measurement of mental distress. The results of the multivariate regression analyses that predicted the outcome variables of UWES and FS-14 by the three latent classes were shown in Table 5. We found significant differences related to fatigue and work engagement among the different classes of mental workload. In particular, nurses in class 3 were significantly more likely to report higher scores of work engagement relative to those in class 2, indicating that nurses who had self-perceived high workload and low self-evaluation were likely to feel more engaged at work compared to nurses who had average workload perception and self-evaluation. Similar patterns were found in the outcomes of FS-14, specifically, nurses in class 3 reported a significantly higher level of fatigue than those in class 2. In other words, the nurses’ workload perception during the COVID-19 outbreak may be positively associated with their fatigue status.

**Table 4:** The mean scores of FS-14 and UWES of mental workload subgroups

	Overall *n* = 1337	Class 1 *n* = 41	Class 2 *n* = 455	Class 3 *n* = 841	*p*
**FS-14**	6.94 ± 2.43	6.68 ± 2.52	6.64 ± 3.12	7.12 ± 3.06	0.045*
physical fatigue	4.89 ± 1.43	4.61 ± 1.53	4.65 ± 1.55	5.04 ± 1.34	0.028*
mental fatigue	2.05 ± 1.18	2.07 ± 1.06	1.99 ± 1.14	2.08 ± 1.09	0.075
**UWES**	57.75 ± 11.47	61.12 ± 12.82	54.43 ± 9.70	59.37 ± 12.02	< 0.001**
vigor	19.59 ± 4.72	20.73 ± 4.02	18.60 ± 3.68	20.07 ± 3.93	< 0.001**
dedication	19.68 ± 4.71	21.07 ± 5.19	18.54 ± 4.22	20.22 ± 3.85	< 0.001**
absorption	18.47 ± 3.96	19.31 ± 4.35	17.29 ± 3.29	19.07 ± 4.16	< 0.001**

Class 1: low workload perception & high self-evaluation group; Class 2: medium workload perception & medium self-evaluation group; Class 3: high workload perception & low self-evaluation group

*p < 0.05; **p < 0.01

**Table 5:** Results of multivariate regressions predicting fatigue and work engagement by mental workload subgroups

	UWES		FS-14	
	B (SE)	*p*	B (SE)	*p*
Class 3 vs. 1	−1.085 (1.283)	0.198	0.200 (0.271)	0.541
Class 3 vs. 2	4.750 (0.892)	< 0.001^**^	0.428 (0.200)	0.033^*^
Class 2 vs. 1	−6.761 (2.285)	0.003^**^	−0.047 (0.575)	0.735

Class 1: low workload perception & high self-evaluation group; Class 2: medium workload perception & medium self-evaluation group; Class 3: high workload perception & low self-evaluation group

B: unstandardized coefficient; *SE* standard error

Adjusted by gender, years in nursing profession, having a bachelor’s degree, marital status, and having children

*p < 0.05; **p < 0.01

**Fig. 2:**
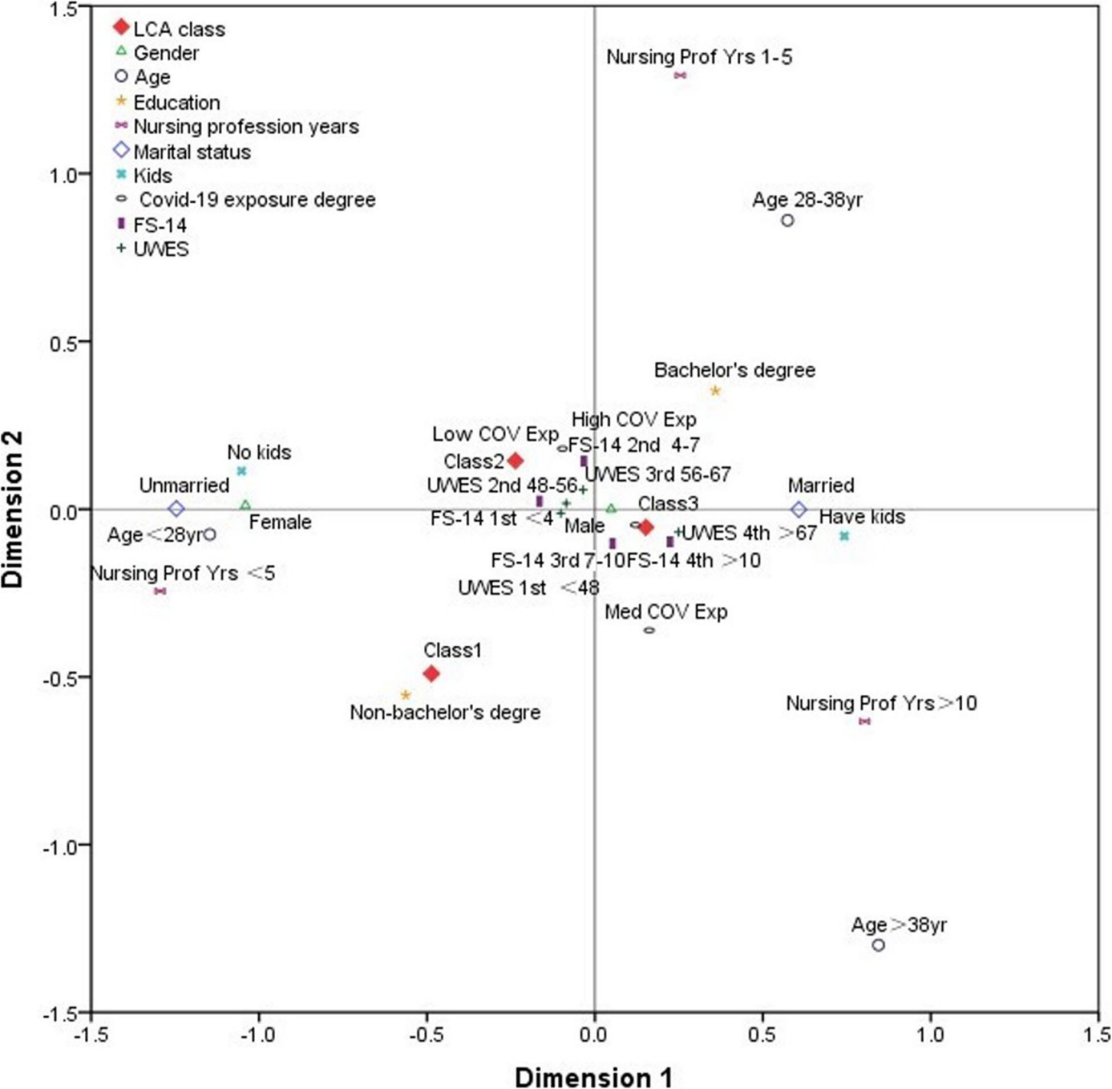
Multiple correspondence analysis plot. The categorical variables entered into the multiple correspondence analysis (MCA) model (based upon optimal scaling) to evaluate the potential association with mental workload. Each variable was visualized with a specific icon and the brief description was labeled near the icon. Interpretation of this graphic output was mainly based on the distance between category-icons

We further entered significant categorical variables into the multiple correspondence analysis (MCA) model to evaluate the potential association among various latent classes. The continuous variables were converted into categorical variables according to the different quartiles (e.g., age, nursing profession year, FS-14, and UWES). In the MCA plot, the distance between any row or column points reflected the similarity (or non-similarity) between the variables. Categorical variables with similar profiles were visualized on a coordinate chart (Fig. 2), which showed that UWES> 67, FS-14 > 7, high & medium COVID-19 exposure, and having children, were associated with class 3; UWES = 48 ~ 67, FS-14 < 7, and low COVID-19 exposure, were correlated with class 2; UWES< 48 and not having a bachelor’s degree, were associated with class 1, respectively.

## Discussion

In this study, we identified three separate mental workload classes among nurses who had worked in hospitals during the COVID-19 outbreak. The three classes can be separated from one another by a relatively low, medium and high workload level. Of the 1337 nurses, the majority (62.9%) belonged to class 3 “high workload perception & low self-evaluation group”, who are most likely to be associated with longer professional years, more fatigue, better work engagement and higher COVID-19 exposure.

Self-perceived stress and mental demands are often underestimated using conventional objective workload indices, such as patient-to-nurse staffing ratios or patient acuity scores. The NASA-TLX is frequently used in emergency and Critical Care Units as a subjective measure of hospital staff workload and is supposed to be a stronger predictor of patient outcomes [23].

Our results showed that nurses’ mental workload score during the COVID-19 outbreak was 77.42 ± 14.82, and it demonstrated higher levels of mental workload than previous studies in terms of the overall score of NASA-TLX [24, 25], especially evident in those in class 3 (mean score of 85.28 ± 0.32), which can be attributed to the heavier overload faced by Chinese nurses during the COVID-19 outbreak. Another characteristic of nurses in class 3 was relatively low self-evaluation (mean score of 14.33 ± 0.28), meaning that they felt more frustrated while working in the hospitals, and this can be partly explained by tense patient-nurse relationships and the higher risk of COVID-19 exposure during the outbreak. During working hours, nurses were required to wear personal protective equipment (PPE) for 6 h or more of work, and coupled with the frequent and time-consuming process of putting on and taking off PPE, would have had adverse effects on the nurses’ mental burden [26].

Comparisons of demographic variables across different subgroups showed that the majority of nurses in class 3 shared the characteristics of older, higher education level, married with child, having more years of clinical practice and working under higher COVID-19 exposure risk, which shows that these individuals were senior professionals who generally took on more challenging and complex nursing tasks. This result is consistent with a previous study that showed employees over 36 years of age had heavier work pressure than those younger than 36 [27]. Older nurses are often imposed with increased responsibility, which may in turn lead to a heavier workload. Nurses in class 1 had the lowest mean mental workload score (mean score of 36.02 ± 2.11) and tended to be younger among the three classes. This might due to the less complexity of clinical practice and technical for novice nurses. However, in contrast to other previous findings [28], our study showed that there were no sex differences between different subgroups, which may be attributed to the substantially lower proportions of male nurses. In order to further assess the impact of the COVID-19 outbreak exposure on nurses’ mental workload, we stratified all subjects to three categories according to the risk of virus exposure across different nursing units. Nurses with the highest exposure risk were those who had direct contact with confirmed COVID-19 positive patients [26]. Group comparison results indicated that COVID-19 exposure contributed to significantly higher mental workload levels. A recent study showed that nurses showed excitability, irritability, unwillingness to rest, and signs of psychological distress in mobile hospitals [29]. Thus, psychological interventions and assistance should be provided to nurses, whether online or face-to-face, in order to help decrease work-related stress.

According to the JD-R model [30], the balance between the job resources and the job demands may lead to higher motivation and positive organizational outcomes, or to the exhaustion of mental and physical energy. Fatigue was recognized as negative outcome, and the work engagement refers to the positive aspect [31]. Previous studies have regarded these three variables as interrelated factors, and mostly used structural equation models to analyze the potential relationship. As suggested by Francesco Pace et al. [9], mental workload leads to fatigue only indirectly through stress and emotional dissonance, while significantly predicting the work engagement among Italian researchers. Similarly, fatigue may be indirectly influenced by mental workload, but also mediated by an individual’s work engagement and their proficiency towards specific tasks [32]. Due to the multidimensional concept of mental workload, the relationships between mental load, fatigue and work engagement are more complicated than that. Our current study showed significant differences in fatigue among nurses belonging to the different subgroups. Multivariate analysis further demonstrated that high cognitive workload significantly increased the subjective assessment of fatigue, regardless of demographic variables such as sex, age, marital state and professional years. This result is in line with a previous study that showed excess cognitive workload resulted in fatigue, independent of inadequate sleep [33]. Theoretically, high mental workloads can result in increased physical fatigue and mental distress in a clinical setting, which may negatively affect nurses’ work engagement [34]. On the contrary, our current study showed that nurses with higher UWES scores belonged to the high workload perception & low self-evaluation group. Thus, there may be a potential positive correlation between nurses’ mental workload and work engagement. Moreover, a positive and supportive working environment can often accommodate nurses’ psychological needs and provide greater work engagement, which requires further investigation.

### Limitations

There are several limitations with the current study. Firstly, a convenience sample was used, and therefore may possibly have limited the generalizability of the study population. Secondly, our study utilized self-reported data exclusively, and therefore may have potential social desirability bias. Thirdly, as a subjective scale, the NASA-TLX has been reported to have lower ecological validity than objective methods for assessing mental workload. In addition, assessment of workload during the working hours may have adverse influence by being perceived as an additional stressor. Thus, we cannot rule out the possible influence of various factors faced by the nurses when filling out the scales. Future studies may opt to assess nurses’ workload by other means, such as workplace observations, supervisor ratings or objective performance indicators.

## Conclusions

The current study examined the mental workload patterns and responses faced by nurses during the COVID-19 outbreak. A total of 62.9% of participants belonged to class 3, that is, those who reported to be the most fatigued. High COVID-19 exposure risk was associated with higher mental workload in nurses. The complex scenario for the care of patients with infectious diseases, especially during an epidemic, raises the need for improved consideration of nurses’ perceived workload, as well as their physical fatigue, work engagement and personal safety. Demographic factors may have variations based on the type of mental workload and may pose a challenge for health institutions in adapting to the changing needs of the environment, and require improved human resource management strategies that are not only patient-centered, but also safe and efficient for nurses.

## Data Availability

The dataset and analyses are not currently publicly available; however, the materials could be available from the corresponding author on reasonable request.

## Abbreviations

COVID-19: Coronavirus disease 2019
SARS-CoV-2: Severe acute respiratory syndrome coronavirus 2
NASA-TLX: National Aeronautics and Space Administration Task Load Index
JD-R: Job demands-resources
LCA: Latent class analysis
FS-14: Fatigue Scale-14
UWES: Utrecht Work Engagement Scale
AIC: Akaike information criterion
BIC: Bayesian information criterion
ABIC: Sample-size adjustment BIC
LMR-LRT: The Lo–Mendell–Rubin likelihood ratio test
ANCOVA: Analysis of covariance
MCA: Multiple correspondence analysis
PPE: Personal protective equipment
